# Biochemical and molecular characterization of Treponema phagedenis-like spirochetes isolated from a bovine digital dermatitis lesion

**DOI:** 10.1186/1471-2180-13-280

**Published:** 2013-12-05

**Authors:** Jennifer H Wilson-Welder, Margaret K Elliott, Richard L Zuerner, Darrell O Bayles, David P Alt, Thad B Stanton

**Affiliations:** 1 Infectious Bacterial Diseases Research Unit, National Animal Disease Center, Ames, Iowa 50010, USA; 2 Food Safety and Enteric Pathogens Research Unit, National Animal Disease Center, Ames, Iowa 50010, USA; 3 Mailing address: USDA, ARS, National Animal Disease Center, 1920 N. Dayton Ave, P.O. Box 70, Ames, IA 50010, USA

**Keywords:** Bovine digital dermatitis, Treponema, Spirochete, Bacterial growth, Genomic comparison

## Abstract

**Background:**

Bovine papillomatous digital dermatitis (DD) is the leading cause of lameness in dairy cattle and represents a serious welfare and economic burden. Found primarily in high production dairy cattle worldwide, DD is characterized by the development of an often painful red, raw ulcerative or papillomatous lesion frequently located near the interdigital cleft and above the bulbs of the heel. While the exact etiology is unknown, several spirochete species have been isolated from lesion material. Four isolates of *Treponema phagedenis*-like spirochetes were isolated from dairy cows in Iowa. Given the distinct differences in host, environmental niche, and disease association, a closer analysis of phenotypic characteristics, growth characteristics, and genomic sequences of *T. phagedenis,* a human genitalia commensal, and the Iowa DD isolates was undertaken.

**Results:**

Phenotypically, these isolates range from 8.0 to 9.7 μm in length with 6–8 flagella on each end. These isolates, like *T. phagedenis*, are strictly anaerobic, require serum and volatile fatty acids for growth, and are capable of fermenting fructose, mannitol, pectin, mannose, ribose, maltose, and glucose. Major glucose fermentation products produced are formate, acetate, and butyrate. Further study was conducted with a single isolate, 4A, showing an optimal growth pH of 7.0 (range of 6–8.5) and an optimal growth temperature of 40°C (range of 29°C-43°C). Comparison of partial genomic contigs of isolate 4A and contigs of *T. phagedenis* F0421 revealed > 95% amino acid sequence identity with amino acid sequence of 4A. *In silico* DNA-DNA whole genome hybridization and BLAT analysis indicated a DDH estimate of >80% between isolate 4A and *T. phagedenis* F0421, and estimates of 52.5% or less when compared to the fully sequenced genomes of other treponeme species.

**Conclusion:**

Using both physiological, biochemical and genomic analysis, there is a lack of evidence for difference between *T. phagedenis* and isolate 4A. The description of *Treponema phagedenis* should be expanded from human genital skin commensal to include being an inhabitant within DD lesions in cattle.

## Background

Bovine papillomatous digital dermatitis (DD) is the primary cause of lameness in dairy cattle and is a growing concern to the beef industry [1]. Lameness attributed to DD costs the producer $125-216/occurrence (treatment, lost productivity) representing a serious financial burden to the farmer, especially when considering that a large percentage of the herd may be affected [2,3]. Typical DD lesions are characterized by a rough, raw raised area most often occurring on the hind limb between the heel bulb and dewclaw and may develop keratinaceous hair-like projections. Lesions appear painful and are prone to bleeding when probed. Lesions generally do not heal spontaneously and may progress to severe lameness. Efficacious vaccines have so far been elusive [4,5]. Despite treatment and attempts at control, reoccurrence of lesions both on the same hoof/cow and within the herd remains high [6]. Additionally, the welfare issue of maintaining food-producing animals in a healthy, pain-free state cannot be ignored [7].

Several *Treponema* species have been identified in tissue biopsies from DD lesions by *in situ* hybridization, immunohistochemistry and 16S rDNA sequence homology [8-12]. Routinely, treponemes are found at the leading edge of lesions, deep within the tissue. Taking into account the spatial distribution of treponemes within the lesion and the robust immune response directed toward them [13-15], it is thought that these organisms may be key factors in DD lesion development.

The goal of this study was to further characterize and compare laboratory growth characteristics, morphology, enzyme profiles, and draft genomic sequences of the *T. phagedenis* DD isolates, originally described by Trott et al. [14]. While these isolates share greater than 98% 16S rDNA homology with *T. phagedenis*, with each other, and with isolates from dairy herds in California [10], the United Kingdom [16], and Sweden [17], antigenic variation and serological reactivity differ [13]. Previous studies have focused on 16S rDNA analysis for phylogenetic relatedness of Treponema isolates. Given differences in environmental niche and host species between DD isolates and *T. phagedenis* type strains, we sought to compare the physical appearance, growth rate, biochemical substrates, and draft genomes. Results of these studies and genome-wide comparisons indicate that *T. phagedenis*-like isolates from DD lesions of cattle are nearly identical to *T. phagedenis*, suggesting an expansion of environmental niches occupied by this bacterium. We propose the description of *T. phagedenis* be expanded to include both human commensal and putative bovine pathogen.

## Results

### Morphology

**Figure 1:**
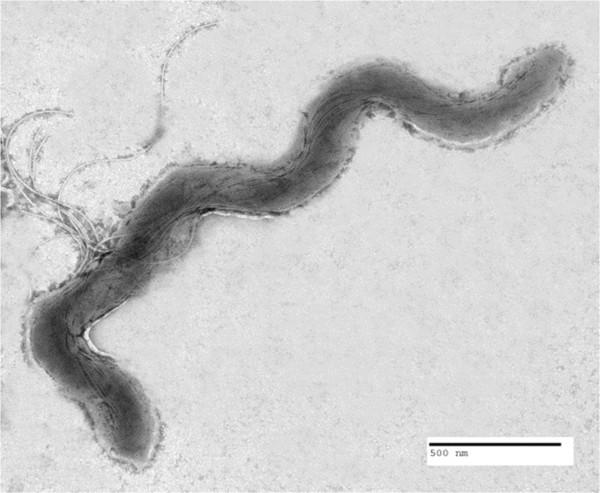
**Negative stained electron photomicrograph of isolate 1A at 13000x magnification showing exposed flagella and insertion disks.** Scale bar equal 500 nm.

Morphological characteristics were determined by phase contrast, dark field, and electron microscopy. Cells were grown in OTI and visualized directly from log-phase culture by phase contrast and dark field microscopy. Cells exhibited typical helical morphology with a slight flattening of the pitch at one or both ends of the cell. Both rotating and translational motility was observed under dark field microscopy. As determined by electron microscopy, cell dimensions of isolates 1A, 3A, 4A and 5B varied from 8 to 9.7 μm in length and 0.3 to 0.35 μm in width, with 7 to 9 flagella attached on terminal ends with 7-14-7, 8-16-8 or 9-18-9 arrangements (Figure 1, Table 1).

**Table 1:** Size and flagella number for Iowa isolates as determined by electron microscopy

	**Isolate 1A**	**Isolate 3A**	**Isolate 4A**	**Isolate 5B**	***T. phagedenis *** **Kazan**
Length (μm) ± StdDev	8.0 ± 0.8	8.7 ± 1.3	9.7 ± 2.6	9.4 ± 0.9	10.4 ± 0.9
Flagella number (single end) ± StdDev	7.3 ± 1.2	7.3 ± 0.5	8.7 ± 0.9	6.6 ± 0.9	6.9 ± 1.2

### API ZYM profile

The enzyme activity profiles of the four Iowa isolates and the reference treponeme species were determined using the API ZYM system. Table 2 shows a comparison of the enzyme activities of these isolates with *T. phagedenis*, *T. denticola,* and other treponeme isolates. The *T. phagedenis*-like DD isolates shared positive reaction for: alkaline phosphatase, C_4_ esterase, C_8_ esterase lipase, acid phosphatase, naptholphosphohydrolase, β-galactosidase, and N-acetyl-β-glucosaminidase. These results matched the *T. phagedenis* biovar Kazan reactivity profile, except that Kazan additionally tested positive for leucine arylamidase activity. The two biovars of *T. phagedenis* (Kazan and Reiter) differed in 6 of the API ZYM tests from each other and are known to differ in enzymatic activity [18]. In contrast, *T. denticola* differed in six different enzymatic reactions from the Iowa DD isolates. Assay variability is clearly demonstrated as in this study *T. denticola* showed positive reactivity for C_8_ esterase lipase, acid phosphatase, naptholphosphohydrolase, α-galactosidase, and α-glucosidase where the same strain published elsewhere was negative for these 5 enzymes but positive for chymotrypsin [19]. Although assay subjectivity and variations in methodology make cross-laboratory comparisons difficult, the API-ZYM profile for Iowa DD isolates closely match the published profile for *T. phagedenis* and *T. brennaborense* as well as several other *T. phagedenis*-like DD isolates including Swedish Bovine isolate V1 [17], isolates from UK cattle Group 2 (*T. phagedenis*-clustering) [16], and several California Bovine isolates [20].

**Table 2:** Comparison of API-ZYM substrate reactivity profiles of Iowa isolates against other DD isolates and known Treponema strains

	**1**	**2**	**3**	**4**	**5**	**6**	**7**	**8**	**9**	**10**	**11**	**12**	**13**	**14**	**15**	**16**	**17**	**18**	**19**
Iowa Isolates 1A, 3A, 4A & 5B*	**+**	**+**	**+**	**-**	**-**	**-**	**-**	**-**	**-**	**+**	**+**	**-**	**+**	**-**	**-**	**-**	**+**	**-**	**-**
*T. phagedenis* Kazan*	+	+	+	-	+	-	-	-	-	+	+	-	+	-	-	-	+	-	-
*T. phagedenis* Reiter§	-	-	-	-	-	-	-	-	-	+	-	-	+	+	-	-	+	-	-
*T. denticola* (ATCC 35405)*	-	+	+	-	-	-	-	+	-	+	+	+	-	-	+	-	-	-	-
*T. denticola* (ATCC 35405) #	-	+	-	-	-	-	-	+	+	-	-	-	-	-	-	-	-	-	-
*T. brennaborense* (isolate DD5/3)§	+	+	+	-	-	-	-	-	-	+	+	-	+	-	+	-	+	-	-
*T. maltophilum* (ATCC 51939)§	+	+	+	-	-	-	-	-	-	+	+	+	-	-	+	-	-	-	+
Bovine isolate V1 & others ¶	+	+	+	-	-^**^	-	-	-	-	+	+	-	+	+	-	-	+	-	-
Isolates from UK cattle, Group 1 (x5)†	+	+	+	-	+	-	-	-	-	+	-	-	-	-	-	-	-	-	-
Isolates from UK cattle, Group 2 (x14)†	+	+	+	-	-	-	-	-	-	+	+	-	+	+	-	-	+	-	+
Isolates from UK cattle, Group 3 (x4)†	-	+	+	-	-	-	-	+	+	-	-	-	-	-	-	-	-	-	-
CA Bovine isolates (x7) ‡	+	+	+	-	-	-	-	-	-	+	+	-	+	+	-	-	+	-	-
Bovine isolate 1-9185MED‡	+	+	+	-	-	-	-	+	+	+	+	-	-	-	-	-	-	-	-

Enzymes: 1, alkaline phosphatase; 2, C_4_ esterase; 3, C_8_ esterase lipase; 4 C_14_ lipase; 5 leucine arylamidase; 6 valine arylamidase; 7 cystine arylamidase; 8, trypsin; 9, chymotrypsin; 10, acid phosphatase; 11, naphtholphosphohydrolase; 12, α-galactosidase; 13, β-galactosidase; 14, β-glucuronidase; 15, α-glucosidase; 16, β-glucosidase; 17, N-acetyl-β-glucosaminidase; 18, α-mannosidase; 19, α-fucosidase.

*As determined in this study, **Isolate T 551B only +.

§ Schrank *et al*. [27], ‡ Walker *et al.*[11], ¶ Pringle *et al*. [17], # Wyss *et al*. [19], † Evans *et al*. [16].

### Volatile fatty acid production

Comparison of metabolite or volatile fatty acid (VFA) production was measured by mass spectrometry of clarified spent medium. Uninoculated medium was incubated similarly to inoculated media and measured for background VFA content. The Iowa DD isolates produced formic, acetic and butyric acids, as did *T. phagedenis* biovar Kazan*.* In contrast, *T. denticola* produced large amounts of acetic and lactic acid but no measurable amount of any other VFA (data not shown).

### Hydrogen sulfide production

All isolates and reference species produced copious amounts of hydrogen sulfide as measured by lead acetate paper suspended above the actively growing culture.

### Substrate utilization and growth conditions

All four of the original Iowa DD isolates shared enzymatic similarity, 16SrRNA gene sequence similarity, and were isolated from the same herd. Consequently, further examination of growth characteristics and nutrient utilization were carried out using isolate 4A. Growth of isolate 4A did not occur in OTI without the addition of bovine rumen fluid or in the absence of volatile fatty acids in BMV (data not shown). Bovine serum was required for growth in both media types. In contrast to *T. vincentii* and *T. denticola*, *T. phagedenis* and isolate 4A required serum in addition to VFA and complex amino acids for growth [21]. Nutrient utilization was determined for isolate 4A cells grown in BMV medium. Isolate 4A grew in the absence of heart infusion broth but growth was restricted in the absence of polypeptone or yeast extract, suggesting an amino acid requirement. Enhanced growth (resulting in an increase in O.D. <0.1 above that seen when isolate 4A was grown in BMV without carbohydrate) was observed using fructose, glucose, maltose, mannitol, mannose, pectin, ribose and soluble starch as carbohydrate source, whereas no enhancement of growth was observed for arabinose, cellobiose, galactose, lactose, sucrose, trehalose or xylose. These results are summarized and compared to two other Treponema species (Table 3). Optimal growth temperature for isolate 4A is 40°C with a range of 29-42°C. Cells in OTI exposed to lower temperatures (down to 4°C) do not grow but remain viable for an extended period of time and will resume growth upon incubation in the optimal temperature range (data not shown). Optimal pH for growth of isolate 4A is pH 7.4 with a range of 6.5-8.0. The general description, temperature, pH range and serum requirement for growth of isolate 4A match those given for *Treponema phagedenis* in Bergey’s Manual of Systematic Bacteriology [18]. Mean generation time in OTI was 4 hours with a maximal density of 10^9^ cells/ml in 96 hours (Additional file 1: Figure S1). Mean generation time in BMV was slightly longer, at 6.8 hours and reaching lower maximal density of 10^8^ cells/ml at 96 hours (Additional file 1: Figure S1).

**Table 3:** Utilization of carbohydrate sources by novel isolate 4A and other known Treponeme species

	Strain 4A**	***T. phagedenis*** **†**	***T. phagedenis *** (ATCC 27087)**	***T. denticola *** **(ATCC 35405)** **	***T. medium*** **†**
Arabinose	-	-	-	-	-
Casein digest	-	nr	-	-	nr
Cellobiose	-	-	-	-	nr
Fructose	+	+	+	-	+
Galactose	-	d	-	-	+
Glucose	+	+	+	-	+
Lactose	-	+	-	-	-
Maltose	+	-	+	-	+
Mannitol	+	+	-	-	-
Mannose	+	+	+	-	+
Pectin	+	nr	+	+	nr
Ribose	+	d	+	-	+
Soluble starch	+	-	+	-	nr
Sucrose	-	-	-	-	+
Trehalose	-	d	+	-	+
Xylose	-	-	-	-	-

† as listed in Bergey’s Manual of Systematic Bacteriology 2^nd^ ED, Vol 4. d- Different biovars give different results, nr- not reported.

**As determined in this study.

### Genomic comparison

Comparisons of proteins predicted for isolate 4A and *T. phagedenis* F0421, whose sequence was obtained from the human microbiome project, made using the RAST server showed a high degree of similarity. At the amino acid level, approximately 86% of the proteins predicted for *T. phagedenis* F0421 demonstrated >95% identity to proteins encoded by genes identified in isolate 4A. Over 50% of the encoded proteins examined demonstrate >99.5% identity (data not shown).

Results from comparisons made using Genome-To-Genome Distance Calculator (GGDC) appear in Table 4. Comparison of genomic contigs from isolate 4A and *Treponema phagedenis* F0421 using either BLAT or BLAST analysis indicate that isolate 4A is >70% similar to F0421 and should not be considered a new species. These comparisons along with the global RAST comparison (4A to F0421) are in agreement that the two isolates are highly similar and should most likely be treated as the same species. Results further indicate that isolate 4A is <70% similar to other fully sequenced *Treponema* species available in Genbank, including *T. succinifaciens, T. azotonutricium, T. primita, T. brennaborense, T. denticola, T. paraluiscuniculi,* and *T. pallidum*.

**Table 4:** **Comparison of Isolate 4A to other treponemes using Genome-To-Genome Distance Calculator (**http://ggdc.gbdp.org/**)**

**Reference Sequence†**	**Comparison Program**	DDH% estimate**
*Treponema phagedenis* F0421* 2.83 Mb, AEFH00000000.1	BLAT	82.11
*Treponema phagedenis* F0421* 2.83 Mb, AEFH00000000.1	NCBI-BLAST	84.59
*Treponema succinifaciens* DSM 2489	"	52.5
Complete chromosome, 2.73 Mb, NC_015385.1		
*Treponema azotonutricium* ZAS 9	"	47.15
Complete chromosome, 3.85 Mb, NC_015577.1		
*Treponema primitia* ZAS 2	"	45.7
Complete chromosome, 4.05 Mb, NC_015578.1		
*Treponema brennaborense* DSM 12	"	35.64
Complete chromosome, 3.05 Mb, NC_015500.1		
*Treponema denticola* ATCC 35405	"	29.34
Complete chromosome, 2.84 Mb, NC_002967.9		
*Treponema paraluiscuniculi* Cuniculi A	"	25.82
Complete chromosome, 1.13 Mb, NC_015714.1		
*Treponema pallidum* subsp. pallidum SS14	"	25.75
Complete chromosome, 1.14 Mb, NC_010741.1		

†All comparisons used 60 Contigs assembled for Isolate 4A as Query and report results using Formula 2 (Identities/HSP length).

**Regression based. DNA-DNA Hybridization (DDH%) estimates ≤70% indicate organisms compared represent different species. Estimates >70% indicate organisms represent same species.

*277 Contigs for Treponema phagedenis F0412 were used as reference sequence.

## Discussion

Treponema spirochetes have been found in many species of animals in close association with their host, with distinct species colonizing genitalia, gastrointestinal tracts and oral cavity. Treponema spirochetes can co-exist as harmless commensals (e.g., *T. refringens, T. minutum*), symbionts of the intestinal tract (e.g., *T. bryantii* of ruminants, *T. primitia* from termites), pathogens (*T. pallidum* spp.) or as part of a pathogenic complex of bacteria (*T. denticola, T. vincentii,* and others from the oral cavity) [20,22]. Additionally, several different phylogenetic groups of *Treponema* species have been isolated or identified in digital dermatitis lesions, with similarities to *T. denticola, T. phagedenis, T. vincentii, T. medium*, and the proposed new species *T. brennaborense* and *T. pedis*[16,23-27]. Four *Treponema* spirochetes were isolated from DD lesions on an Iowa dairy, and the characterization presented here demonstrates that they are highly similar to the *T. phagedenis* type strain. Despite classification as the same genus, these organisms occupy not just different hosts (bovine vs. human), but also very different anatomical locations (dermis adjacent to heel bulb and dewclaw vs. genitalia). There most likely exists some overlap of microenvironment within these anatomical locations (low oxygen availability, epithelial cell layers, etc.) as both the DD isolates and *T. phagedenis* have similar growth characteristics and nutrient requirements.

Other pathogenic organisms such as *Mycobacterium intracellulare*, *Yersinia* species and *Bacillus* species have identical 16 s rRNA gene sequences and are highly genetically similar based on DNA-DNA hybridization [28]. However, they exhibit distinct “ecophysiological” properties based on virulence phenotypes or host ranges. Some are distinct species, *Y. pestis* and *Y. pseduotuberculosis* for example, while others are merely different serovars within the species, such as *M. intracellulare*. Some pathogens are separated from other genetically identical species by acquisition of a plasmid conferring pathogenic properties. Evaluation of the draft contigs of *T. phagedenis* and the DD isolates do not give any indication of acquisition of a plasmid that would have conferred the expansion of host range or conversion into a more virulent organism.

These studies herein led us to develop a growth medium reduced in complexity so that the individual nutrients and growth factors of previously isolated spirochetes could be further evaluated. While the list of components appear similar to fastidious anaerobe broth used by many groups [17,29], the quantities of several components are greatly reduced. Systematic studies on essential nutrients and environmental growth factors of the non-pallidum treponemes are scarce [22] and consist of a few incomplete lists in such reference texts as Bergey’s Manual of Systematic Bacteriology and The Prokaryotes [18,21]. A recently published report showed that isolate 1A achieved log phase growth in 3 to 5 days of culture in a rich media similar to fastidious anaerobe broth [29] consistent with our results in both media types.

We have defined temperature tolerances, pH tolerances and essential growth requirements (serum and VFAs) of isolate 4A. It was very interesting that an organism isolated from the hoof of a cow was tolerant to and preferred higher temperatures (up to 40°C). The hoof temperature of a dairy cow ranges from 21 to 23°C [30]. The hoof surface temperature was found to increase in cases of DD, sole ulcers, or other hoof diseases [30], and thus could create a more favorable environment for treponemal growth.

Further insight into the Iowa DD isolates physiology was sought by evaluation of substrate utilization and enzymatic activity of the treponeme isolates. By understanding growth requirements and nutritional capabilities of these isolates, we can begin to piece together the microenvironment necessary for optimal survival and growth of the treponemes. As in the case of human periodontal disease, one bacterial colonizer may provide the nutritional substrates for secondary colonizers and tissue destructive bacteria [31]. There were little differences between *T. phagedenis* and the DD isolates on the basis of enzymatic activity or substrate utilization, mainly regarding mannitol and trehalose. While there were slight differences in enzymatic profiles, these are generally not sufficient for the separation into different species. For example, *T. phagedenis* biovar Reiter is able to hydrolyze esculin but biovar Kazan does not [18]. As the complete sequences of both *T. phagedenis* and these DD isolates become available, these small biochemical differences may be explained by alterations in the genome consistent with host adaptation.

Past studies have evaluated the similarity of DD *Treponema* isolates based on sequencing of 16S ribosomal regions, 16-23S intergenic spacer regions or conserved flagellin genes (i.e., *flaB2*). Previously published work has shown that the *T. phagedenis*-like isolates 9–3301, 7–2009, 2–1498 from California, and 1A and 4A from Iowa, have >99% identical 16S-23S rRNA gene sequence and intergenic spacer regions clustered into the same phylotype based on product length polymorphisms [10]. Although a completed genome for any *T. phagedenis* isolate is not available, comparison of assembled contigs for isolate 4A revealed a high degree of similarity throughout the genome. Differences in the number of genes identified (3251 in isolate 4A and 2799 genes in F0421) most likely reflect a difference in sequencing coverage and completeness of the resulting contigs. Performance of *in silico* DDH using isolate 4A and F0421 further supports classification of the bovine lesion isolates as *T. phagedenis*.

## Conclusion

These results indicate that a similar bacterium has been independently isolated in several geographical locations (*i.e.*, IA, CA, Sweden, UK, Germany) but also from bovine and human hosts. However, even with the high degree of genomic, structural, and physiological similarity between isolates, variation exists with regard to immune reactivity and host recognition of differing surface antigens [13,32]. In conclusion, the bovine isolates are by all tests nearly identical to *T. phagedenis* biovar Kazan and published sequences of *T. phagedenis* reference tp_F0421 and as such do not represent novel species. The descriptions of *T. phagedenis* should be expanded to describe the organism as human genitalia commensal and putative pathogen of bovine digit.

## Methods

### Bacterial cultures

Type species *Treponema phagedenis* bivar Kazan (ATCC 27087), *Treponema vincentii* LA (ATCC 35580) and *Treponema denticola* (ATCC 35405) were purchased from the American Type Culture Collection (ATCC, Manassas, VA). *T. phagedenis*-like ioslates 1A, 3A, 4A and 5B were isolated from lesions on Iowa dairy cattle as previously described [14].

### Culture media and conditions

Treponeme isolates were cultured in two different media for these studies: oral Treponeme isolation (OTI) broth and basal minimal media with volatile fatty acids (BMV). Media were prepared under 100% nitrogen as previously described [14] and formulas are listed (Table 5). As needed, 15 g per L noble agar (DIFCO) and 5% bovine blood were added. BMV was formulated to grow spirochetes in a minimal nutrient medium and facilitate metabolic end product analyses. Cultures were adapted to BMV for at least five passages before being utilized in analyses. All studies were conducted using cultures under anaerobic atmosphere conditions (5% hydrogen, 5% carbon dioxide, 90% nitrogen) in chemically reduced media. Optimal pH for growth of isolate 4A was determined by using OTI and adjusting the pH using 1 N hydrochloric acid or 1 N sodium hydroxide. Growth substrates were identified by adding different carbohydrate sources to BMV media (Table 5). Bacterial density was measured using a spectrophotometer and related to bacterial cell numbers as determined from direct cell counts using dark field microscopy.

**Table 5:** Composition of oral Treponema isolation (OTI) and basal minimal media with VFA (BMV) media used in these studies

**Component**	**OTI**	**BMV**
Polypeptone	5.0 g	5.0 g
Heart Infusion Broth	5.0 g	5.0 g
Yeast Extract (YE)	5.0 g	1.0 g
Glucose	0.8 g	†
Pectin	0.8 g	†
Soluble Starch	0.8 g	†
Arabinose		†
Casein Digest		†
Cellobiose		†
Fructose		†
Mannitol		†
Galactose		†
Lactose		†
Trehalose		†
Mannose		†
Sucrose	0.8 g	†
Maltose	0.8 g	†
Ribose	0.8 g	†
Xylose	0.8 g	†
Sodium Pyruvate	0.8 g	†
K_2_HPO_4_	2.0 g	2.0 g
NaCl	5.0 g	5.0 g
MgSO_4_	0.1 g	0.1 g
Cysteine-HCl	0.68 g	1.0 g
DI Water	500 ml	822 ml
Resazurin (0.1%)	1.0 ml	1.0 ml
Rumen Fluid	500 ml	
VFA Solution**		10 ml
Bovine Serum§	1 ml/10 ml	1 ml/10 ml

† - To test carbohydrate substrates 0.5 ml of a 10% solution of each was added to 8.5 ml media just before reduction and inoculation.

**VFA Solution consisted of 0.5 ml each of isovaleric, isobutyric, n-valeric, and DL-a-methylbutyric acid in 100 ml 0.1 N NaOH.

§ Final concentration = 10% Bovine serum, added to 8.5 ml medium just before reduction and inoculation.

### Electron microscopy

Actively dividing cells of the DD isolates were grown in OTI and were prepared for transmission electron microscopy. Cells were collected by centrifugation (10,000 × g, 10 minutes) and washed twice in cold 10 mM phosphate buffered saline (pH 7.4, PBS) with final suspension in distilled water. Samples were negatively stained with an equal volume of 2% phosphotungstic acid (pH 7.0) and mounted on a formvar/carbon reinforced 200-mesh copper grid. Grids were examined at 80 kV under a FEI Tecnai G2 electron microscope equipped with AMT camera.

### Metabolic characterization

Bacterial cells from log phase culture grown in BMV with glucose and 10% bovine serum were collected by centrifugation (10,000 × g, 10 minutes), washed twice in isotonic saline and resuspended in isotonic saline to a density of 5–6 using McFarland standard. The API-ZYM test (bioMerieux) was performed per manufacturer’s instructions. The enzyme β-glucosidase (0.2 g/L, Sigma) was used as an internal control.

### Volatile fatty acid quantification

To determine volatile fatty acid production, 9.9 mL of BMV medium with glucose and 10% bovine serum was inoculated with 100 μl of 1 × 10^8^ growing bacterial cells/ml and incubated at 37°C for 72–96 hrs. The culture was then centrifuged to remove cellular material and the supernatant prepared for gas-phase liquid chromatography as previously described [33-35]. Uninoculated medium was used as a control.

### Hydrogen sulfide production

100 μl containing 1 × 10^8^ bacterial cells/ml from log phase cultures were inoculated into 9.9 ml BMV and cultured for 72 hours. Hydrogen sulfide was assayed by using the lead acetate test as previously described [36].

### DNA sequencing and analysis

DNA from isolate 4A was extracted from 100 mL growing broth cultures using DNeasy Blood and Tissue Kit (Qiagen, Valencia, CA) as per manufacturer’s instructions. Sequencing reactions were based upon Roche FLX-Titanium and Titanium + chemistry (Roche/454 Life Sciences, Branford, CT 06405; http://www.454.com) as well as Illumina chemistry (Illumina, Inc., San Diego, CA 92122; http://www.illumina.com). Genomic DNA was processed according to manufacturer’s instructions for preparation of DNA libraries. Whole genome random libraries were prepared and sequenced using the Illumina HiSeq 2000 and a Roche GS-FLX + instrument. In addition, genomic DNA was used to prepare paired-end libraries of 2Kb and 8Kb according to Roche protocols and was sequenced using the Roche GS-FLX + instrument and Titanium sequencing chemistries. Sequencing data from each of the methodologies was used to perform a *de novo* assembly using both the MIRA assembler [37] and the Roche gsAssembler (Newbler) version 2.6, (Roche/454 Life Sciences, Branford, CT 06405, USA; http://www.454.com) Mauve Genome Alignment software was employed to compare assemblies and optimize the resulting *de novo* assembly. The draft genome assembly consisted of 42 contigs in 14 scaffolds and a total of 3,027,773 bp assembled (Newbler) from a combined coverage of greater than 90×. This Whole Genome Shotgun project has been deposited at DDBJ/EMBL/GenBank under the accession AQCF00000000. The version described in this paper is the first version, AQCF01000000.

Assembled contigs for 4A and for *Treponema phagedenis* F0421 (277 contigs, http://www.ncbi.nlm.nih.gov/Traces/wgs/?val=AEFH01; Accession: PRJNA47285ID: 47285, Accession: PRJNA62291ID: 62291, AEFH00000000.1) from the human microbiome project were submitted to the National Microbial Pathogen Data Resource (NMPDR), SEED-based, Rapid Annotation using Subsystems Technology (RAST) server [38] for annotation and comparison. Isolate 4A was chosen as the reference for RAST comparison purposes, as the genome assembly was in fewer total contigs. A total of 3251 genes were identified in isolate 4A and 2799 genes in F0421. Proteins predicted from the annotated sequence were examined at various levels of percent amino acid identity.

Assembled contigs for 4A were submitted for comparison using the Genome-To-Genome Distance Calculator (GGDC) (http://ggdc.gbdp.org/) [39].

Comparison of isolate 4A and *Treponema phagedenis* F0421 genomes was additionally performed using Blast-Like Alignment Tool (BLAT) [40] because it corresponds better to actual DNA-DNA hybridization (DDH) comparisons than do comparisons performed using BLAST. For genomes that are not closed, (i.e. a file of assembly contigs such as is the case for both 4A and F0421), only Formula 2 results should be relied upon for making determinations [41]. Isolate 4A was also compared to at least one representative of other *Treponema* species available in Genbank for a total of 8 comparisons. Since only one genome was closed in these subsequent analyses, the comparisons were based on Basic Local Alignment Search Tool (BLAST) results. DDH-based speciation of bacterial isolates is based on a limit of 70% similarity in order to make a determination of a new species. Genomes ≤70% similar should be considered different species while genomes >70% similar indicate they should not be considered a new species [41].

## Competing interest

The authors declare they have no competing interests.

## Authors’ contributions

MKH, RLZ conceived the study, designed and inititated biochemical and biological experimental work. JHWW completed experimental biochemical and biological work, prepared manuscript for publication. RLZ, DPA, DOB designed, implemented and performed sequencing, cloning, and genomic analysis experiments. TBS provided critical insight and guidance for research and manuscript preparation. All authors contributed to, read and approved the final manuscript.

## Supplementary Material

Additional file 1: Figure S1 Comparison of growth rate for isolate 4A in OTI and BMV. After 5 sequential passages in either OTI or BMV, 1 × 10^7^ mid-log phase cells were inoculated in to 10 ml OTI or BMV and absorbance measured over time. Results are representative of 3 independent experiments, and error bars indicate standard error of the mean. Click here for file
