# Supplementary material for: Biochemical and molecular characterization of Treponema phagedenis-like spirochetes isolated from a bovine digital dermatitis lesion
Source: BMC Microbiol. 2013 Dec 5;13:280. doi: 10.1186/1471-2180-13-280 (PMC3879011; doi:10.1186/1471-2180-13-280)
Supplement: Additional file 1: Figure S1 — Comparison of growth rate for isolate 4A in OTI and BMV. After 5 sequential passages in either OTI or BMV, 1 × 10^7^ mid-log phase cells were inoculated in to 10 ml OTI or BMV and absorbance measured over time. Results are representative of 3 independent experiments, and error bars indicate standard error of the mean. [file 1471-2180-13-280-S1.docx]

Additional file 1: Figure S1. Comparison of growth rate for isolate 4A in OTI and BMV. After 5 sequential passages in either OTI or BMV, 1x10^7^ mid-log phase cells were inoculated in to 10 ml OTI or BMV and absorbance measured over time. Results are representative of 3 independent experiments, and error bars indicate standard error of the mean.
